# Association between TLR2 and TLR4 Gene Polymorphisms and the Susceptibility to Inflammatory Bowel Disease: A Meta-Analysis

**DOI:** 10.1371/journal.pone.0126803

**Published:** 2015-05-29

**Authors:** Yang Cheng, Yun Zhu, Xiuping Huang, Wei Zhang, Zelong Han, Side Liu

**Affiliations:** 1 First clinical college, Southern Medical University, Guangzhou, Guangdong Province, China; 2 Liver Tumor Center, Nanfang Hospital, Southern Medical University, Guangzhou, Guangdong Province, China; 3 Department of Digestion, Nanfang Hospital, Southern Medical University, Guangzhou, Guangdong Province, China; Indiana University School of Medicine, UNITED STATES

## Abstract

**Background:**

The associations between toll-like receptor 2 (TLR2) and toll-like receptor 4(TLR4) polymorphisms and inflammatory bowel disease (IBD) susceptibility remain controversial. A meta-analysis was performed to assess these associations.

**Methods:**

A systematic search was performed to identify all relevant studies relating TLR2 and TLR4 polymorphisms and IBD susceptibility. Odds ratios (ORs) and 95% confidence intervals (CIs) were calculated. Subgroup analyses were performed by ethnicity and publication quality.

**Results:**

Thirty-eight eligible studies, assessing 10970 cases and 7061 controls were included. No TLR2 Arg677Trp polymorphism was found. No significant association was observed between TLR2 Arg753Gln polymorphism and Crohn’s disease (CD) or ulcerative colitis (UC) in all genetic models. Interestingly, TLR4 Asp299Gly polymorphism was significantly associated with increased risk of CD and UC in all genetic models, except for the additive one in CD. In addition, a statistically significant association between TLR4 Asp299Gly polymorphism and IBD was observed among high quality studies evaluating Caucasians, but not Asians. Associations between TLR4 Thr399Ile polymorphisms and CD risk were found only in the allele and dominant models. The TLR4 Thr399Ile polymorphism was associated with UC risk in pooled results as well as subgroup analysis of high quality publications assessing Caucasians, in allele and dominant models.

**Conclusions:**

The meta-analysis provides evidence that TLR2 Arg753Gln is not associated with CD and UC susceptibility in Asians; TLR4 Asp299Gly is associated with CD and UC susceptibility in Caucasians, but not Asians. TLR4 Thr399Ile may be associated with IBD susceptibility in Caucasians only. Additional well-powered studies of Asp299Gly and other TLR4 variants are warranted.

## Introduction

Inflammatory bowel disease (IBD), which mainly consists of ulcerative colitis (UC) and Crohn's disease (CD), is a group of chronic non-specific gastrointestinal inflammatory conditions. IncreasedIBD incidence and prevalence havebeen observed in different regions of the world[1]. IBD is an autoimmune disease that results from an aberrant immune response to intestinal bacteria or other foreign substances as well as genetic factors[2]. Previous studies have demonstrated that genetic polymorphisms contribute to individual variations in the genetic susceptibility to IBD[3]. Among the genetically predisposing alleles tightly linked to IBD, Toll-like receptor (TLR) polymorphisms have attracted increasing attention in recent years[4].

Toll-like receptors (TLRs) are transmembrane proteins usually expressed by antigen presenting cells; they are important immune receptors that participate in the recognition of pathogen-associated molecular patterns and activation of signal transduction pathways of antimicrobial genes, by identifying and binding to small molecular components on pathogens[5]. TLRs also play an important role in the digestive system. In addition, they can recognize invading microbes in the intestinal barrier and activate the immune response. However, sustained hyper-activation of TLRs may lead to chronic inflammation in IBD. At present, at least 13 TLR family members recognizing different pathogens independently or together in various internal organs have been described, of which TLR2 and TLR4 are most commonly studied for their association with risk of IBD[6].

TLR2, located at 4q31.3, recognizes bacterial lipopeptides and lipoteichoic acid found abundantly in the cell wall of Gram positive bacteria[7]. TLR4, located at 9q33.1, serves as a surface receptor for lipopolysaccharides (LPS), the main endotoxins derived from Gram-negative bacteria[8]. In the normal intestine, TLR2 and TLR4 are expressed at low levels in intestinal epithelial cells (IECs), thus minimizing the recognition of luminal bacteria[9]. However, TLR2 and TLR4 are up-regulated in primary IECs throughout the lower gastrointestinal tract in IBD patients, which may cause excessive immune response[10–12].

Population-based case-control show an association between TLR4 polymorphism and susceptibility to CD and UC. The association between TLR2 gene variantss and extensive colonic disease in UC and CD has also been discribed[13]. TLR2 Arg677Trp (R677W, rs12191786) and Arg753Gln (R753Q, rs5743708), and TLR4 Asp299Gly (D299G, rs4986790) and Thr399Ile (T399I, rs4986791) polymorphisms are the most widely discussed SNPs in the investigation of the association between polymorphisms of TLR family and susceptibility to IBD. The association between TLR4 Asp299Gly and Thr399Ile polymorphisms and IBD is controversial. TLR2 single studies did not found the association of TLR2 Arg677Trp and Arg753Gln polymorphisms and IBD. However, these studies has relatively sample size and might be underpowered to reveal a small effect of the polymorphisms of TLR2 on IBD susceptibility. Meta-analysis can combine results from different studies to produce an estimate of the major effect with enhanced precision.The aim of this meta-analysis was to investigate the associations between TLR2 (Arg677Trp, Arg753Gln) and TLR4 (Asp299Gly, Thr399Ile) genetic polymorphisms and susceptibility to IBD.

## Methods

### Literature search

A systemic search was conducted on PubMed, Embase, Biosis Preview and China National Knowledge Infrastructure databases up to August 31, 2014 using the following keywords: (1) “toll-like receptor” or “TLR”; (2) “Crohn’s disease” or “CD” or “ulcerative colitis” or “UC” or “inflammatory bowel disease” or “IBD”; (3) “polymorphism” or “variant” or “genotype”. There was no language restriction and species were limited to human. References in the reviews and retrieved articles were hand-searched as well. For articles by the same author using the same case series, the study with the largest sample size was selected.

### Inclusion criteria

The inclusion criteria were: (1) case-control study; (2) investigation evaluating the relationship between TLR2 (Arg677Trp, Arg753Gln) and TLR4 (Asp299Gly, Thr399Ile) genetic polymorphisms and IBD (CD or UC) susceptibility; (3) sufficient available published data for odds ratio (OR) estimation with 95% confidence interval (CI); (4) human study; (5) data not republished.

### Data extraction and quality assessment

Missing data were requested by contacting study authors through email. Data were blindly extracted from all selected publications by two investigators (Cheng and Zhu) separately. For each of the included articles, the first author name, publication year, study population (ethnicity), source of controls, total numbers of patients and controls, and polymorphism frequencies in patients and controls were extracted. For studies that included subjects of different ethnic groups, data were extracted for each one. Any disagreement on a given item of the extracted data was fully discussed to reach a consensus.

Predefined criteria (Table 1) based on the scale of Thakkinstian[14] were used to assess the methodological quality of eligible studies. The revised criteria cover the representativeness of cases and controls, assessment of IBD, genotyping examination, Hardy-Weinberg equilibrium (HWE) in the control population, and association assessment. Scores ranged from 0 (lowest) to 11 (highest). Articles with scores of less than 6 were considered to be low-quality studies, whereas those with scores equal to or higher than 6 were considered high-quality reports. Quality assessment was also performed by two authors separately (Yang and Yun). Disagreements were resolved by consensus as well.

**Table 1:** Scale for methodologic Quality Assessment of the Single Nucleotide Polymorphism association studies of IBD.

	Criteria	Score
A	**Representativeness of cases**	
	Consecutive/randomly selected from case population with clearly defined sampling frame	2
	Consecutive/randomly selected from case population without clearly defined sampling frame or with extensive inclusion/exclusion criteria	1
	No method of selection described	0
B	**Representativeness of controls**	
	Controls were consecutive/randomly drawn from the same sampling frame (ward/community) as cases	2
	Controls were consecutive/randomly drawn from a different sampling frame as cases	1
	Not described	0
C	**Ascertainment of IBD**	
	Clearly described objective criteria for diagnosis of IBD	2
	Diagnosis of IBD by patient self-report or by patient history	1
	Not described	0
D	**Genotyping examination**	
	Genotyping done under “blinded” condition	1
	Unblinded or not mentioned	0
E	**Hardy-Weinberg equilibrium**	
	Hardy-Weinberg equilibrium in control group	2
	Hardy-Weinberg disequilibrium in control group	1
	No checking for Hardy-Weinberg equilibrium	0
F	**Association assessment**	
	Assess association between genotypes and IBD with appropriate statistics and adjustment for confounders	2
	Assess association between genotypes and IBD with appropriate statistics without adjustment for confounders	1
	Inappropriate statistics used	0

### Statistical analysis

Pooled crude odds ratios (ORs) and 95% confidence intervals (95% CIs) were determined to assess the associations between TLR2 (Arg677Trp, Arg753Gln) and TLR4 (Asp299Gly, Thr399Ile) genetic polymorphisms and the risk of IBD under dominant, recessive, additive and allele models, based on the extracted data. The fixed-effects (random-effects) model was used depending on the heterogeneity among studies[15, 16]. Subgroup analysis was performed to assess the ethnic-specific effects. Potential heterogeneity was examined by the chi-square based Q-test and I^2^. A P value for heterogeneity < 0.10 or I^2^ > 50% was considered statistically significant. Sensitivity analysis was performed by sequentially excluding each single study to assess the stability of the results[17]. Galbraith plots were performed to identify possible distinct articles, which might contribute to the heterogeneity[18]. Hardy-Weinberg equilibrium (HWE) in the control group was assessed by the chi-square test[19]. Potential publication bias was estimated by the funnel plot of the ORs versus their standard errors[20]. Funnel plot asymmetry was assessed by the Egger’s test (linear regression test) when the number of studies included was more than 10. P value > 0.10 indicated no significant publication bias[21]. Studies with P value <0.1 were corrected using the Duval’s trim and fill method[22]. All statistical analyses were performed with STATA 10.0 (StataCorp LP, College Station, TX).

## Results

### Literature search

**Fig 1:**
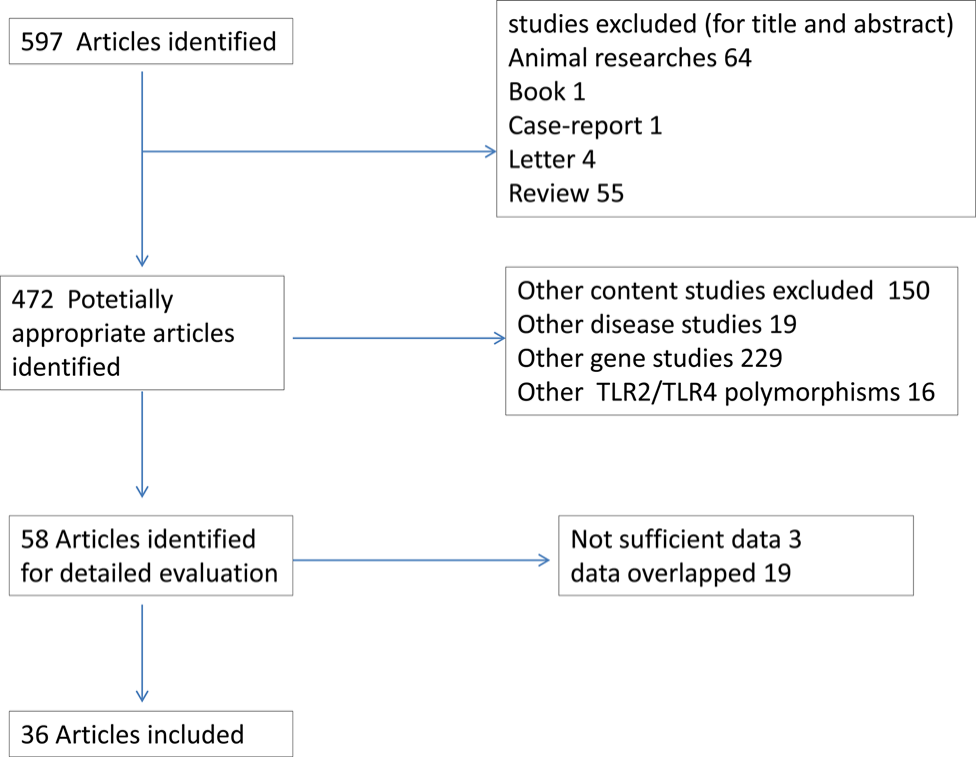
Flow chart showing literature search for studies of TLR2 and TLR4 polymorphism in relation to risk of CD and UC.

This meta-analysis was performed and reported according to the PRISMA guidelines. The search of PubMed, Biosis Previews, Embase and two Chinese databases (Chinese National Knowledge Infrastructure and Wanfang databases) for relevant articles published up to July 2014 yielded 597 articles. A total of 36 articles[23–58][23–58] met the inclusion criteria and were selected. Three article contained 2 separated studies each, as each of them involved two different populations. Overall, 39 studies, assessing 10970 cases and 7061 controls were included in the analysis. A flow chart demonstrating the selection process of relevant studies is represented in Fig 1.

### Study characteristics and quality assessment

The basic information for each study, including authors and publication years, ethnicity, numbers of cases and controls, frequencies of various genotypes in IBD patients and healthy controls, and Hardy-Weinberg equilibrium (HWE) in healthy controls, are summarized in Table 2. Of the 39 qualifying studies, 23 were conducted among Caucasian populations, 10carried out among Asians and 6 performed in other ethnicities. All studies were published between 2002 and 2013, and were case-control designed. IBD patients and controls were age and gender matched in 6 studies, while the other 33 studies did not specifically mention this detail in their reports. Allelic distribution for TLR2 and TLR4 is shown in S1 Table.

**Table 2:** Characteristics of the included studies on TLR polymorphism and susceptibility of CD and UC.

	Year of	Author	Region	TLR	Phenotype	Cases			Controls			HWE in
	Publication			Variant	studied	Number	Males(%)	Age	Number	Males(%)	Age	controls
1	2002	Okayama[23]	Japanese	TLR4 299	UC	UC: 86	nr	nr	107	nr	nr	equilibrium
2	2004	Arnott[24]	Scotland	TLR4 299	CD&UC	CD: 234	43.7	28(21–41)	189	52.2	38(27–50)	equilibrium
						UC: 246	53.4	33(25–49)				equilibrium
3	2004	Franchimont(1)[25]	Belgium	TLR4 299	CDs&UC	CD: 334	40.7	26.6±10.3	139	nr	nr	equilibrium
						UC: 163	52.2	29.78±12.8				
4	2004	Franchimont(2)[25]	Belgium	TLR4 299	CD	CD: 114	56.2	28.9±12.4	139	nr	nr	equilibrium
5	2004	Torok[26]	Germany	TLR4 299	CD&UC	CD: 102	36.3	40.9±13.7	145	49	44.6±12.5	equilibrium
				TLR4 399		UC: 98	45.9	42.7±13.3				
6	2005	Brand [27]	Germany	TLR4 299	CD	CD: 204	47.1	37.8±11.8	199	49.8	46.4±15.3	equilibrium
				TLR4 399								
7	2005	Fries[28]	Italy	TLR4 299	CD	CD:23	56.5	43(15–75)	59	47.5	38(18–68)	nr/ equilibrium
8	2005	Gazouli[29]	Greek	TLR4 299	CD&UC	CD: 120	nr	nr	100	nr	nr	nr/disequilibrium
				TLR4 399		UC: 85	nr	nr				
9	2005	Ouburg[30]	The Netherlands	TLR4 299	CD	CD:112	nr	nr	170	nr	nr	nr/equilibrium
10	2005	Braat[31]	The Netherlands	TLR4 299	CD&UC	CD: 441	35%	mean 40.7	137	nr	nr	equilibrium
						UC: 226	53%	mean 44.4				
11	2005	Oostenbrug[32]	The Netherlands	TLR4 299	CD&UC	CD: 393	nr	nr	296	nr	nr	equilibrium
				TLR4 399		UC: 179	nr	nr				
12	2005	Lakatos[33]	Hungary	TLR4 299	CD	CD:527	50.3	37.1±7.6	200	0.51	38.05±10.7	nr/ equilibrium
13	2006	Figueroa[34]	Chile	TLR4 299	CD&UC	CD: 22	36.4	46.8(16–65)	20	nr	nr	nr/imponderable
						UC: 22	40.9	37.8(19–67)				
14	2006	Pierik[35]	Belgium	TLR2 753	CD&UC	CD:179	41.1	25.0±10.1	191	nr	nr	equilibrium
				TLR4 299		UC:106	54.3	28.9±13.3				
15	2007	Xiong[36]	China	TLR2 677	IBD	120	nr	nr	110	nr	nr	nr/imponderable
				TLR2 753								
				TLR4 299								
				TLR4 399								
16	2007	Jiang[37]	China	TLR4 299	UC	UC:68	57.4	37.58±12.37	152	57.9	46.90±12.73	equilibrium
17	2007	Xue[38]	China	TLR2 677	CD&UC	CD: 41	60.7	34. 54±14. 21	135	55.6	41. 85±10. 82	nr/imponderable
				TLR2 753		UC: 43		45. 95±17. 11				
				TLR4 299								
				TLR4 399								
18	2007	Henckaerts[39]	Belgium	TLR2 753	CD&UC	CD: 874	41	24 (18–31)	312	45	39(30–57)	equilibrium
				TLR4 299		UC: 259	52	26 (21–36)				
19	2007	Hong[40]	New Zealand	TLR2 753	CD	CD:182	nr	nr	188	nr	nr	equilibrium
				TLR4 299								
				TLR4 399								
20	2007	Baumgart(1)[41]	Hungary	TLR4 299	CD&UC	CD: 144	43.1	24±11.2	202	46.5	(18–54)	nr/ equilibrium
						UC: 118	37.3	31±10.6				
21	2007	Baumgart(2)[41]	Germany	TLR4 299	CD&UC	CD: 235	38.3	26±10.3	403	42.4	(21–61)	nr/ equilibrium
						UC: 145	46.2	31±13.6				
22	2007	Browning[42]	New Zealand	TLR4 299	CD&UC	CD: 389	36	nr	416	44	nr	equilibrium
				TLR4 399		UC: 405	47	nr				
23	2008	Rigoli[43]	Italy	TLR4 299	CD&UC	CD:133	52.6	43.5 ± 10.7	103	66	46.6 ± 9.8	equilibrium
				TLR4 399		UC:45	60	43.2 ± 11.0				
24	2008	Hume[44]	Australia	TLR4 299	CD&UC	CD:619	nr	nr	360	nr	nr	equilibrium
						UC:300	nr	nr				
25	2008	Akin[45]	Turkey	TLR4 299	CD&UC	CD:108	nr	nr	191	52.4	35.2 ±11.2	nr/ equilibrium
				TLR4 399		UC:120	nr	nr				
26	2008	Lappalainen[46]	Finland	TLR4 299	CD&UC	CD: 240	nr	nr	190	nr	nr	equilibrium
				TLR4 399		UC: 459	nr	nr				
27	2009	Ye[47]	Korea	TLR4 299	CD	CD: 380	62.6	27.2±7.7	380	52.3	36.6±13.8	nr/ disequilibrium
28	2009	Zouiten-Mekki[48]	Tunisia	TLR4 299	CD&UC	CD:90	nr	nr	80	nr	nr	nr/ disequilibrium
				TLR4 399		UC:30	nr	nr				
29	2009	Queiroz[49]	Brazil	TLR2 677	CD&UC	CD:43	46.51	40.88±14.16	541	75.6	33.87±9.96	equilibrium
				TLR2 753		UC:42	14.29	38.93±14.73				
				TLR4 299								
30	2009	Bueno[50]	Belgium	TLR4 299	CD&UC	CD: 80	60	22.86±7.4	79	nr	nr	equilibrium
						UC: 15	58.8	18.3±5.3				
31	2010	Wagner[51]	Australia	TLR4 299	CD	CD: 72	63.9	11.6(2.2–17.2)	98	45.9	11.9 (1.7–19.8)	equilibrium
32	2010	Shen[52]	China	TLR2 677	CD&UC	CD:30	60	32.5(14–64)	120			equilibrium
				TLR2 753		UC:83	60.2	46.0(19–72)				
				TLR4 299								
				TLR4 399								
33	2011	Chen(1)[53]	China	TLR2 677	CD&UC	CD:30	nr	nr	60	49	36.8 ± 12.2	equilibrium
				TLR2 753		UC:40	nr	nr				
				TLR4 299								
34	2011	Chen(2)[53]	China	TLR2 677	CD&UC	CD:30	nr	nr	84	48.5	33.2 ± 12.0	equilibrium
				TLR2 753		UC:46	nr	nr				
				TLR4 399								
35	2012	Sivaram[54]	India	TLR4 299	UC	UC: 139	nr	nr	176	nr	nr	nr/ equilibrium
36	2012	Azzam[55]	Saudi	TLR4399	CD	CD:46	67.4	30.43 ± 10.20	50	nr	nr	nr/ equilibrium
37	2012	Kim[56]	Korea	TLR2 677	CD&UC	CD: 45	56	32.1±11.4	178	49	47.2±13.0	equilibrium
				TLR2 753								
				TLR4 299		UC: 99	46	49.8±14.7				
				TLR4 399								
38	2012	Guagnozzi[57]	Italia	TLR4 299	CD&UC	CD: 84	nr	nr	227	nr	nr	nr/ equilibrium
						UC: 133	nr	nr				
39	2013	Manolakis[58]	Greece	TLR4 299	CD&UC	CD: 187	47.8	43.23±22.9	274	55.2	46.9±22.4	equilibrium
				TLR4 399		UC: 163	63.1	50.1±18.6				

Of the 39 studies, 7 assessed the TLR2 Arg677Trp polymorphism, 10 studied the TLR2 Arg753Gln polymorphism, 37 evaluated the TLR4 Asp299Gly polymorphism and 17 studied the TLR4Thr399Ile polymorphism. There were 30 high quality and 9 low quality studies, respectively according to the set quality criteria (Table 3). All low quality studies encompassed TLR4 polymorphism analyses.

**Table 3:** Quality assessment of studies included.

	Year of	Aurthor	Representativeness	Representativeness	Ascertainment	Genotyping	Hardy-Weinberg	Association	Total
	Publication		of cases	of controls	of IBD	examination	equilibrium	assessment	
1	2002	Okayama	0	0	0	0	2	1	3
2	2004	Torok	0	0	2	0	2	1	5
3	2004	Arnott	2	0	2	0	2	1	7
4	2004	Franchimont(1)	2	2	2	0	2	1	7
5	2004	Franchimont(2)	2	2	2	0	2	1	7
6	2005	Brand	0	0	2	0	2	1	5
7	2005	Fries	2	2	1	0	0	1	6
8	2005	Gazouli	2	2	2	0	0	1	7
9	2005	Ouburg	2	2	2	0	0	1	7
10	2005	Braat	2	2	2	0	2	1	9
11	2005	Oostenbrug	2	2	2	0	2	1	9
12	2005	Lakatos	2	0	2	0	0	1	5
13	2006	Figueroa	2	2	2	0	0	1	7
14	2006	Pierik	2	2	1	0	2	0	7
15	2007	Xiong	2	2	2	0	0	1	7
16	2007	Jiang	2	2	2	0	2	1	9
17	2007	Xue	2	2	2	0	2	1	9
18	2007	Henckaerts	2	2	2	0	2	0	8
19	2007	Hong	1	1	2	0	2	1	7
20	2007	Baumgart(1)	1	1	2	0	0	1	5
21	2007	Baumgart(2)	1	1	2	0	0	1	5
22	2008	Lappalainen	1	1	2	0	2	1	7
23	2007	Browning	2	1	2	0	2	1	8
24	2008	Rigoli	2	2	2	0	2	1	9
25	2008	Hume	2	1	2	0	2	1	8
26	2008	Akin	2	2	1	0	0	1	6
27	2009	Ye	2	2	2	0	0	1	7
28	2009	Zouiten-Mekki	0	0	2	0	0	1	3
29	2009	Queiroz	2	2	2	0	2	2	8
30	2009	Bueno	2	2	2	0	2	1	9
31	2010	Wagner	2	1	2	0	2	1	8
32	2010	Shen	2	2	2	0	2	1	9
33	2011	Chen(1)	2	2	2	0	2	1	9
34	2011	Chen(2)	2	2	2	0	2	1	9
35	2012	Sivaram	0	0	2	0	0	1	3
36	2012	Azzam	2	2	2	0	0	1	7
37	2012	Kim	2	2	2	0	2	1	9
38	2012	Guagnozzi	0	0	1	0	0	1	2
39	2013	Manolakis	2	1	2	0	2	1	8

### Main meta-analysis results

**Fig 2:**
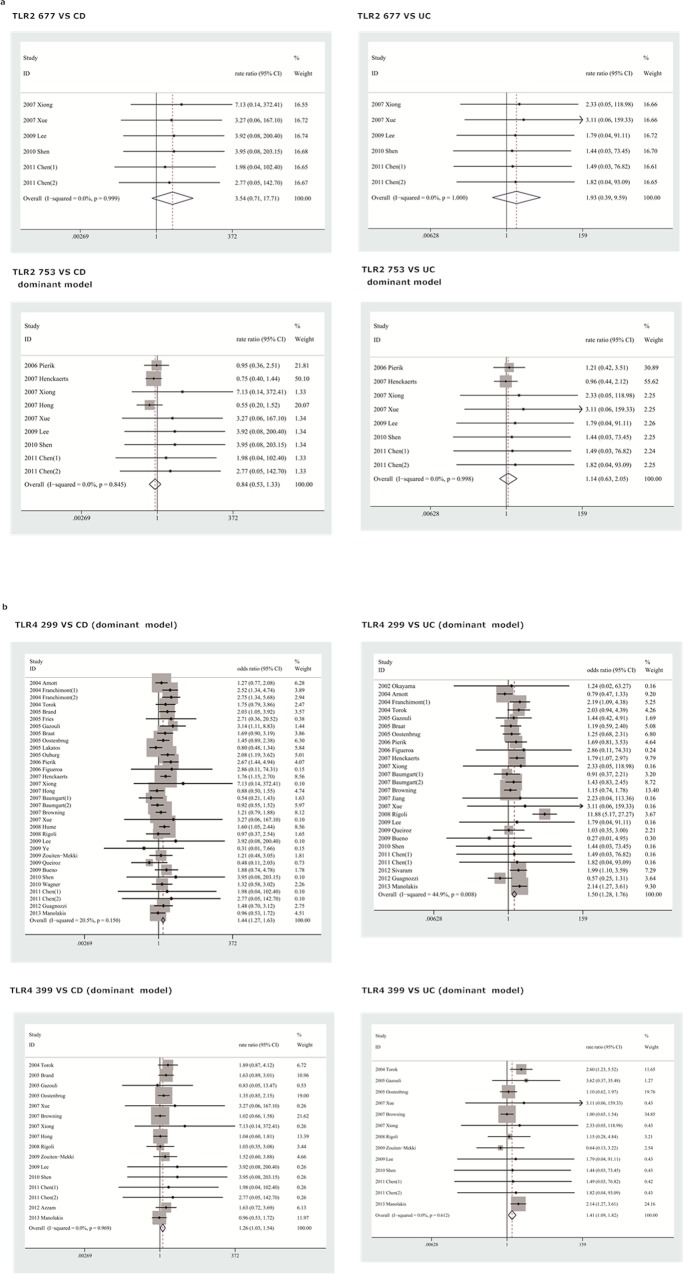
Forest plot showing the association between TLR2 and TLR4 polymorphisms and CD and UC risk. Squares represent the effect size for the odds ratios of CD or UC risk among subjects. Error bars represent 95% confidence intervals (CI). Diamonds represent pooled estimates within each analysis. (a) TLR2 polymorphisms and CD/UC in dominant model; (b) TLR4 polymorphisms and CD/UC in dominant model.

An estimation of the association between TLR2 Arg677Trp, Arg753Gln and TLR4Asp299Gly, Thr399Ile polymorphisms and susceptibility to CD and UC is presented in Table 4. The corresponding forest plots are shown in Fig 2.

**Table 4:** Results of the meta-analysis of the relationship of TLR2 and TLR4 polymorphism with CD or UC risk.

Study	Genetic model	OR	95% CI	I2(%)	P value	No. of study	Egger
TLR2 677 vs CD	dominant model	3.54	0.71–17.71	0.00	0.99	6	/
	additive model	3.54	0.71–17.71	0.00	0.99	6	/
	recessive model	3.54	0.71–17.71	0.00	0.99	6	/
TLR2 677 vs UC	dominant model	1.93	0.39–9.59	0.00	1.00	6	/
	additive model	1.93	0.39–9.59	0.00	1.00	6	/
	recessive model	1.93	0.39–9.59	0.00	1.00	6	/
TLR2 753 vs CD	dominant model	0.84	0.53–1.33	0.00	0.84	9	/
	allele model	2.69	0.77–9.44	0.00	1.00	9	/
TLR2 753 vs UC	dominant model	1.14	0.63–2.05	0.00	1.00	8	/
	allele model	1.14	0.63–2.05	0.00	1.00	8	/
TLR4 299 vs CD	dominant model	1.44	1.27–1.63	20.50	0.15	33	0.50
	additive model	1.62	0.98–2.67	0.00	1.00	32	0.39
	recessive model	1.82	1.11–3.01	0.00	1.00	32	0.74
	allele model	1.40	1.24–1.57	25.00	0.10	33	0.49
TLR4 299 vs UC	dominant model	1.50	1.28–1.76	44.90	0.01	26	0.82
	additive model	2.37	1.29–4.35	0.00	1.00	26	0.97
	recessive model	2.25	1.22–4.12	0.00	1.00	26	0.91
	allele model	1.40	1.22–1.62	43.60	0.01	27	0.62
TLR4 399 vs CD	dominant model	1.26	1.03–1.54	0.00	0.97	16	0.04
	additive model	1.45	0.66–3.18	0.00	0.98	16	0.59
	recessive model	1.35	0.62–2.95	0.00	0.98	16	0.41
	allele model	1.21	1.01–1.44	0.00	0.95	17	0.10
TLR4 399 vs UC	dominant model	1.41	1.09–1.82	0.00	0.61	13	0.56
	additive model	1.89	0.70–5.13	0.00	1.00	13	0.74
	recessive model	1.84	0.68–5.00	0.00	1.00	13	0.65
	allele model	1.26	1.02–1.56	0.00	0.52	14	0.36
**High quality studies**							
TLR4 299 vs CD	dominant model	1.56	1.35–1.80	3.00	0.42	26	0.47
	additive model	1.72	0.98–3.02	0.00	1.00	25	0.44
	recessive	1.94	1.10–3.40	0.00	1.00	25	0.60
	allele model	1.50	1.31–1.72	12.20	0.29	26	0.47
TLR4 299 vs UC	dominant model	1.55	1.28–1.87	49.10	0.01	20	0.66
	additive model	2.49	1.25–4.95	0.00	0.99	20	0.97
	recessive	2.35	1.18–4.67	0.00	0.99	20	0.88
	allele model	1.51	1.16–1.98	47.10	0.01	21	0.46
TLR4 399 vs CD	dominant model	1.16	0.92–1.46	0.00	0.98	13	0.03
	additive model	1.50	0.65–3.46	0.00	0.92	13	0.53
	recessive	1.40	0.61–3.21	0.00	0.92	13	0.37
	allele model	1.13	0.93–1.37	0.00	0.96	14	0.14
TLR4 399 vs UC	dominant model	1.32	1.00–1.75	0.00	0.78	11	0.38
	additive model	1.65	0.55–4.90	0.00	1.00	11	0.43
	recessive	1.62	0.54–4.81	0.00	1.00	11	0.41
	allele model	1.19	0.95–1.49	0.00	0.76	12	0.24
**Group by ethinicity**							
TLR2 753 vs CD							
Asia	dominant model	3.54	0.71–17.71	0.00	0.99	6	/
	allele model	3.54	0.71–17.71	0.00	0.99	6	/
Caucasian	dominant model	0.73	0.36–1.47	0.00	0.48	2	/
	allele model	1.05	0.07–16.82	0.00	0.99	2	/
TLR2 753 vs UC							
Asian	dominant model	1.93	0.39–9.59	0.00	1.00	6	/
	allele model	1.93	0.39–9.59	0.00	1.00	6	/
Caucasian	dominant model	1.05	0.56–1.97	0.00	0.77	2	/
	allele model	1.50	0.09–24.07	0.00	0.87	2	/
TLR4 299 vs CD							
Asian	dominant model	2.17	0.52–9.14	0.00	0.92	7	/
	additive model	2.17	0.52–9.14	0.00	0.92	7	/
	recessive	2.17	0.52–9.14		0.92	7	/
	allele model	1.87	0.45–7.74	0.00	0.80	7	/
Caucasian	dominant model	1.45	1.28–1.64	37.90	0.04	23	/
	additive model	1.72	1.00–2.96	0.00	1.00	23	/
	recessive	1.74	1.01–2.99	0.00	1.00	23	/
	allele model	1.43	1.26–1.62	42.50	0.02	22	/
Others	dominant model	0.99	0.46–2.10	0.00	0.46	3	/
	additive model	0.98	0.10–9.61	0.00	1.00	3	/
	recessive	2.17	0.22–21.12	0.00	0.57	3	/
	allele model	1.14	0.78–1.66	0.00	0.64	4	/
TLR4 299 vs UC							
Asian	dominant model	1.86	0.46–7.46	0.00	1.00	8	/
	additive model	1.86	0.46–7.46	0.00	1.00	8	/
	recessive	1.86	0.46–7.46	0.00	1.00	8	/
	allele model	1.86	0.46–7.46	0.00	1.00	8	/
Caucasian	dominant model	1.51	1.01–2.07	67.90	0.00	15	0.93
	additive model	2.14	1.04–4.39	0.00	0.98	15	0.86
	recessive	1.99	0.97–4.08	0.00	0.99	15	0.82
	allele model	1.48	1.11–1.96	64.10	0.00	15	0.94
Others	dominant model	1.73	1.04–2.88	0.00	0.55	3	/
	additive model	8.10	1.17–55.93	0.80	0.37	3	/
	recessive	7.74	1.12–53.40	4.90	0.35	3	/
	allele model	1.20	0.88–1.64	47.60	0.13	4	/
TLR4 399 vs CD							
Asian	dominant model	3.54	0.71–17.71	0.00	1.00	6	/
	additive model	3.54	0.71–17.71	0.00	1.00	6	/
	recessive	3.54	0.71–17.71	0.00	1.00	6	/
	allele model	3.57	0.72–17.75	0.00	1.00	6	/
Caucasian	dominant model	1.20	0.97–1.49	0.00	0.78	8	/
	additive model	0.98	0.30–3.21	0.00	0.78	8	/
	recessive	0.96	0.29–3.14	0.00	0.78	8	/
	allele model	1.19	0.96–1.46	0.00	0.62	8	/
Others	dominant model	1.58	0.85–2.93	0.00	0.91	2	/
	additive model	1.28	0.32–5.11	0.00	0.87	2	/
	recessive	1.07	0.28–4.16	0.00	0.92	2	/
	allele model	1.20	0.85–1.69	0.00	0.70	3	/
TLR4 399 vs UC							
Asian	dominant model	1.93	0.39–9.59	0.00	1.00	6	/
	additive model	1.93	0.39–9.59	0.00	1.00	6	/
	recessive	1.93	0.39–9.59	0.00	1.00	6	/
	allele model	1.93	0.39–9.59	0.00	1.00	7	/
Caucasian	dominant model	1.42	1.09–1.85	43.70	0.11	6	/
	additive model	1.80	0.47–6.92	0.00	0.85	6	/
	recessive	1.71	0.44–6.59	0.00	0.84	6	/
	allele model	1.42	1.11–1.83	38.10	0.15	6	/
Others	allele model	0.91	0.62–1.35	0.00	0.67	2	/

### TLR2 Arg677Trp

All studies evaluating the TLR2 Arg677Trp polymorphism were conducted among Asians. No variant allele A carrier or mutant homozygous was found in either the IBD patients or control population in the included studies. In addition, TLR2 Arg677Trp polymorphism did not show any association with CD (OR = 3.54, 95%CI = 0.71–17.71, P = 0.99) or UC (OR = 1.93, 95%CI = 0.39–9.60, P = 1.00) in Asian populations.

### TLR2 Arg753Gln

Due to the rarity of the TLR2 Arg753Gln mutant homozygous genotype in the included studies, the data could only be pooled in the allele and dominant models. In the allele model, no association was found between the A allele and CD(A vs G: OR = 2.69, 95%CI = 0.77–9.44, P = 1.00)or UC(A vs G: OR = 1.81, 95%CI = 0.45–7.26, P = 1.00)susceptibility. Similarly, the AA genotype was not associated with risk of CD (AA vs GG: OR = 0.84, 95%CI = 0.53–1.82, P = 1.00) or UC (AA vs GG: OR = 1.14, 95%CI = 0.63–2.05, P = 1.00).

Subgroup analyses based on ethnic were performed to assess CD and UC susceptibility in Asians and Caucasians. No significant association was identified in both Asians(for CD, A vs G: OR = 3.54, 95%CI = 0.71–17.71, P = 0.99; AA vs GG: OR = 3.54, 95%CI = 0.71–17.71, P = 0.99; for UC, A vs G: OR = 1.93, 95%CI = 0.39–9.59, P = 1.00; AA vs GG: OR = 1.93, 95%CI = 0.39–9.59, P = 1.00) and Caucasians (G vs. A: OR = 1.05, 95% CI: 0.07–16.82, P = 0.99; AA vs. GG: OR = 0.73, 95% CI: 0.36–1.47, P = 0.48).

### TLR4 Asp299Gly

A significantly increased susceptibility was found between TLR4 D299G and CD in the allele model (A vs G: OR = 1.40, 95%CI = 1.24–1.57, P = 0.1), dominant model (AA+GA vs GG: OR = 1.44, 95%CI = 1.27–1.63, P = 0.15), recessive model (AA vs GA+GG: OR = 1.82, 95%CI = 1.11–3.01, P = 1.00) and additive model (AA vs GG: OR = 1.62, 95%CI = 0.98–2.67, P = 1). Similar results were also found between TLR4 D299G and UC in the allele model (A vs G: OR = 1.40, 95%CI = 1.22–1.62, P = 0.01), dominant model (AA+GA vs GG: OR = 1.50, 95%CI = 1.28–1.76, P = 0.01), recessive model (AA vs GA+GG: OR = 2.25, 95%CI = 1.22–4.12, P = 1.00) and additive model (AA vs GG: OR = 2.37, 95%CI = 1.29–4.35, P = 1.00).

Stratified analyses by study quality and ethnicity were conducted to further explore the actual effect of TLR4 D299G polymorphism on the risk of CD and UC. Similar results were obtained with subgroup analyses by study quality. When ethnicity was restricted to Asians, no TLR4 D299G polymorphism was found in patients with CD or UC. However, when only studies with Caucasians were considered, a significant association with CD was obtained in all contrast models (A vs G: OR = 1.43, 95%CI = 1.26–1.62, P = 0.02; AA vs GG: OR = 1.72, 95%CI = 1.00–2.99, P = 1; AA+GA vs GG: OR = 1.45, 95%CI = 1.28–1.64, P = 0.04; AA vs GA+GG: OR = 1.74, 95%CI = 1.01–2.99, P = 1.00). Furthermore, significant associations with UC were found in the allele model (A vs G: OR = 1.48, 95%CI = 1.11–1.96, P<0.01), dominant model (AA+GA vs GG: OR = 1.51, 95%CI = 1.10–2.07, P = P<0.01) and additive model (AA vs GG: OR = 2.14, 95%CI = 1.04–4.39, P = 0.98). However, in the recessive model, only a marginal association (AA vs GA+GG: OR = 1.99, 95%CI = 0.97–4.08, P = 0.99) was found. Similar results were found in UC in Caucasians (A vs G: OR = 1.48, 95%CI = 1.11–1.96, P<0.01; AA vs GG: OR = 2.14, 95%CI = 1.04–4.39, P = 0.98; AA+GA vs GG: OR = 1.51, 95%CI = 1.10–2.07, P<0.01; AA vs GA+GG: OR = 1.99, 95%CI = 0.97–4.08, P = 0.99).

### TLR4 Thr399Ile

The pooled results of all studies suggested that TLR4 T399I polymorphism was significantly associated with CD susceptibility in the dominant (TT+CT vs CC: OR = 1.26, 95%CI = 1.03–1.54, P = 0.97) and allele (T vs C: OR = 1.21, 95%CI = 1.01–1.44, P = 0.95) models, whereas no significant association was found in the recessive (TT vs CT+CC: OR = 1.35, 95%CI = 0.62–2.95, P = 0.98) and additive (TT vs CC: OR = 1.45, 95%CI = 0.66–3.18, P = 0.98) models. Similarly, the CC genotype significantly increased UC susceptibility in the dominant model (TT+CT vs CC: OR = 1.41, 95%CI = 1.09–1.82, P = 0.61) and the C allele was found associated with a higher UC susceptibility in the allele model (T vs C: OR = 1.26, 95%CI = 1.02–1.56, P = 0.52); no significant association was found in the recessive (TT vs CT+CC: OR = 1.84, 95%CI = 0.68–5.00, P = 1.00) and additive (TT vs CC: OR = 1.89, 95%CI = 0.70–5.13, P = 1.00) models.

Meta-analyses of high quality studies showed that TLR4 T399I polymorphism was not associated with the risk of CD in any genetic model and only the dominant model showed a significant association with the risk of UC.

Stratified by ethnicity, neither Asians nor Caucasians were associated with CD susceptibility in all four genetic models. With respect to UC, a significant association was found for Caucasians in dominant (TT+CT vs CC: OR = 1.42, 95%CI = 1.09–1.85, P = 0.11) and allele (T vs C: OR = 1.42, 95%CI = 1.11–1.83, P = 0.15) models, but not in recessive (TT vs CT+CC: OR = 1.71, 95%CI = 0.44–6.59, P = 0.84) and additive (TT vs CC: OR = 1.80, 95%CI = 0.47–6.92, P = 0.85) models.

### Heterogeneity analysis

**Fig 3:**
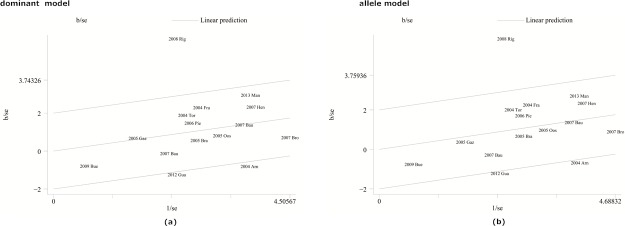
Galbraith plot of the association between TLR4 299 polymorphism and UC risk in Caucasians. Each figure represents a unique article in this meta-analysis. The figures outside the three lines were spotted as the outlier and the possible source of heterogeneity in the analysis pooled from the total available numbers. (a) Galbraith plot results of TLR4 299 polymorphisms and UC risk in the dominant model; (b) Galbraith plot results of TLR4 299 polymorphisms and UC risk in the allele model.

For the TLR4 299 polymorphism versus UC, a statistically significant heterogeneity among studies was found in the dominant and allele models with the I^2^ values of heterogeneity> and P values< 0.10. To further investigate the heterogeneity in studies assessing TLR4 299 polymorphism in Caucasians, Galbraith plots were generated to identify the outliers which might contribute to this observation. Our results showed that the study published in 2008 by Rigoli was an outlier in both dominant and allele models (Fig 3). All I^2^ and P values decreased overtly after excluding 2008 Rigoli in dominant and allele models with Caucasians. The heterogeneity of the remainingmeta-analyses was acceptable.

### Sensitivity analysis

Sensitivity analysis was performed by sequentially excluding individual studies. For analyses pooling more than three individual studies, the summary ORs were not influenced by excluding any single study (data not shown), indicating that our results were statistically robust.

### Publication bias

**Fig 4:**
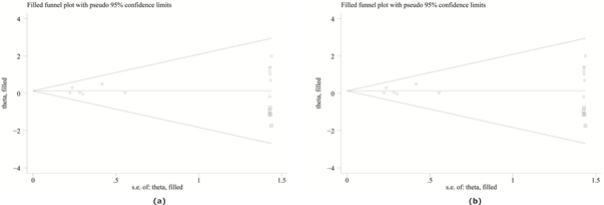
Funnel plots for studies evaluating TLR4 399 polymorphisms and risk of CD included in the meta-analysis. (a) Trim and fill data for all studies on TLR4 299 polymorphisms and UC risk in the dominant model; (b) Trim and fill data for high quality studies on TLR4 299 polymorphisms and UC risk in the dominant model. Imputed data (squares) are imaginary values to compensate for non-symmetric funnel plot.

There was no evidence of obvious asymmetry in the funnel plots. The Egger’s test was performed to access publication bias in the articles included in this meta-analysis, when the number of included studies was greater than 10. All p values obtained in the Egger’s test were more than 0.1 except for the dominant model of TLR4 399 in CD for both overall analysis and the assessment including only high quality studies. There were five unreported studies according to the Duval’s trim and full method (Fig 4). After possibly unpublished studies were imputed, the pooled OR and 95%CI were slightly shifted toward null (Overall study: OR = 1.24, 95%CI = 1.02–1.51; High quality study: OR = 1.13, 95%CI = 0.91–1.42). However, no change was observed in the meta-analysis results.

## Discussion

Genome-wide association studies(GWAS) has improved our knowledge of many common variants and molecular pathways leading to IBD[59]. Recently, a meta-analyses of GWAS conducted by Jostins et al. have identified 163 loci that are significantly associated with IBD[60]. Such discoveries are limited to studies in North America, Oceania and Europe. Yang, S.K., et al. conducted a GWAS and two validation studies in the Korean population and revealed three new susceptibility loci for CD[61]. Till now, most GWAS were conducted in Caucasians with limited studies in other populations. Despite the success of GWAS in identifying IBD susceptibility loci, it explains only a minority of(<25%) the variance in IBD risk[62]-. The advent of GWAS also prompt mechanistic research aimed at exploring the complex interplay between genes, immune networks, and microbiome. Functional studies to assess the in vivo impact of the genetic variants involved in IBD also emerges. Recently, Coelho, T et al. performed a unique systematic review of literature with mechanistic studies in assessing the functional impact of the a selected panel of gene variants implicated in IBD through GWAS and other genetic studies. However, they limited to only 71 genes and TLR is not included in the study. They did not make a meta-analysis due to the lack of functional studies and more functional studies is needed[63].

Both TLR4 and TLR2 was not detected to be associated with IBD in the previous GWAS. Not surprisingly, uncommon genetic variation which may contribute significantly toward the heritability of IBD may not be captured by GWAS[64]. Population-based studies have also provided compelling evidence for genetic factors contribute to the IBD for these rarer variants are more likely to be population specific. We performed a meta-analysis of population based case-control studis for TLR2 and TLR4 polymorphism and IBD susceptibility.

All studies assessing the TLR2 Arg677Trp polymorphism and IBD were carried out in Asia, and no TLR2 Arg677Trp polymorphism was found in Asians, as described above. This is the first meta-analysis evaluating TLR2 Arg677Trp polymorphism and IBD.

In the case of TLR2 Arg753Gln, we only pooled data in the allele and dominant models due to the rarity of the TLR2 Arg753Gln mutant homozygous genotype in the included studies. All studies were of high quality. Our meta-analysis showed no association between the Arg753Gln polymorphism and UC or CD. We then restricted to ethnicity-specific data for subgroup analyses, and found that the Arg753Gln polymorphism was not associated with UC or CD susceptibility in Asians or Caucasians. These findings indicated that Arg753Gln with the mutant allele does not significantly increase IBD susceptibility.To our knowledge, this is the first meta-analysis evaluating TLR2 Arg753Gln polymorphism and IBD.

Interestingly, this meta-analysis revealed a modest association between the TLR4 Asp299Gly polymorphism and IBD (CD and UC). This result was very well supported: sensitivity analyses excluding low quality studies did not significantly change the magnitude of the gene effect or genetic model. Next, we restricted to race-specific assessments to perform a subgroup analysis. Consistent with meta-studies reported by Hume and Shen[44, 52], our study suggested that TLR4 Asp299Gly polymorphism might be associated with UC and CD susceptibility in Caucasians. Browning et al found no relationship between TLR4 Asp299Gly and UC in Caucasians, which contradicts our findings[42]. However, only 12 articles were included in their study. Some differences exist between our study and Hume and Shen’s reports. First, the included studies were updated. Then, quality assessment and publication bias assessment of the included studies were performed. TLR4 Asp299Gly polymorphism was not associated with UC or CD in Asians in our study. Ng et al. has made a systematic review and meta-analysis of genetics of IBD in Asia and they also found no association of TLR4 Asp299Gly and risk of CD[65].However, they only included two studies (Xiong 2006 and Ye 2009) conducted in Chinese and Korean patients. We included 7 more studies with 1 conducted in Japanese, 1 in Korean and 5 in Chinese. Our study might further confirm that population differences, such as genetic heterogeneity, play a vital role in IBD susceptibility.

The current study indicates that the TLR4 399T allele might increase the risk of UC and CD. It is plausible that TLR4 Thr399Ile’s T allele affect TLR4 transcription and expression, further impacting TLR4 protein function. Further studies should focus on how the variant might impact gene expression and function. Subgroup analysis of high quality studies showed no association between TLR4 Thr 399Ile polymorphism and CD, and marginal association between TLR4 Thr399Ile polymorphism and UC. In the meta-analysis performed by Shen et al[52], TLR4 399 Ile polymorphism is associated with both UC and CD susceptibility in Caucasians. Our race-specific subgroup analyses found that the TLR4 Thr 399Ile polymorphism was associated with UC susceptibility in Caucasians while no association between TLR4 Thr399Ile polymorphism and CD susceptibility was observed. For Asians, there was no association observed in any genetic model for TLR4 Thr399Ile and CD or UC susceptibility.

The overall meta-analysis has little heterogeneity. When ethnicity sub-stratification was performed, heterogeneity was decreased or even removed among Asians while significant heterogeneity existed in study for TLR4 Asp299Gly polymorphism and UC in the dominant and allele models in Caucasions. Galbraith plot was used to identify heterogeneous records. One heterogeneous article for TLR Asp299Gly vs UC was detected by the Galbraith plot[43]. The potential bias of the article might result from the elderly population assessed or unknown reasons. After omitting this article, heterogeneity decreased substantially and the association was still significant. The Egger’s test suggested that there was no significant publication bias except for the meta-analysis of TLR4 Thr399Ile and CD in the dominant model. This publication bias was then corrected using the Duval’s trim and fill method. Publication bias is caused by the tendency of researchers and editors to publish reports with positive results, while those showing inconclusive results are likely not considered for publication. Several original studies has controls depart from the HWE which may cause bias in estimates of genetic effects results. Currently, there is no consensus on whether to pool studies that are not in HWE for meta-analysis of genetic association studies. We performed a quality assessment of the studies based on the HWE as well as other criteria such as representativeness of cases and controls. Sensitive analysis quality were also performed by excluding low quality studies in our study which is the merit of our study.

A few limitations of this study need to be mentioned. First, a variety of confounding factors may be associated with increased damage to IBD, such as gender, age, smoking status, clinical phenotype, et al. Unfortunately, we were unable to obtain sufficient data to perform appropriate stratified analyses due to the limited information in the included studies. In addition, the number of cases and controls included was relatively small and most were included in Asians as far as studies on TLR2 Arg677Trp and Arg753Gln are concerned. Thus, the association between in different populations need to be confirmed by further studies. Finally, we could not identify the gene-gene and gene-environment interactions in this study. In conclusion, TLR2 Arg677Trp and Arg753Gln is not associated with the risk of UC or CD. The TLR4 Asp299Gly and Thr399Thr genotypes seem to be more susceptible to UC and CD in Caucasian populations but not in Asians. This finding needs to be further confirmed in future well-designed studies including different ethnicities.

## Supporting Information

S1 PRISMA Checklist PRISMA checklist. (DOC)

S1 Table Allelic distribution for TLR2 and TLR4. (DOCX)

S2 Table Studies referring TLR2/TLR4 polymorphism and IBD susceptibility, but not meet the inclusion criteria for the meta-analysis. (DOC)
