# Supplementary material for: Association between TLR2 and TLR4 Gene Polymorphisms and the Susceptibility to Inflammatory Bowel Disease: A Meta-Analysis
Source: PLoS One. 2015 May 29;10(5):e0126803. doi: 10.1371/journal.pone.0126803 (PMC4449210; doi:10.1371/journal.pone.0126803)
Supplement: S1 Table — (DOCX) [file pone.0126803.s002.docx]

**Table S1 Allelic distribution for TLR2 and TLR4**

| TLR2 Arg677Trp | |  |  |  |
| --- | --- | --- | --- | --- |
|  |  | control | CD | UC |
| 2004 Guo | GG | 110 | 15 | 47 |
|  | GA | 0 | 0 | 0 |
|  | AA | 0 | 0 | 0 |
| 2010 Shen | GG | 120 | 30 | 83 |
|  | GA | 0 | 0 | 0 |
|  | AA | 0 | 0 | 0 |
| 2009 Queiroz | GG | 530 | 43 | 41 |
|  | GA | 11 | 0 | 1 |
|  | AA | 0 | 0 | 0 |
| 2009 Lee | GG | 178 | 45 | 99 |
|  | GA | 0 | 0 | 0 |
|  | AA | 0 | 0 | 0 |
| 2011 Chen（1） | GG | 60 | 30 | 40 |
|  | GA | 0 | 0 | 0 |
|  | AA | 0 | 0 | 0 |
| 2011 Chen（2） | GG | 84 | 30 | 46 |
|  | GA | 0 | 0 | 0 |
|  | AA | 0 | 0 | 0 |
| 2007 Xue | GG | 135 | 41 | 43 |
|  | GA | 0 | 0 | 0 |
|  | AA | 0 | 0 | 0 |
|  |  |  |  |  |

| TLR2Arg753Gln | | |  | |  | | |  | | |  | | |  | | |
| --- | --- | --- | --- | --- | --- | --- | --- | --- | --- | --- | --- | --- | --- | --- | --- | --- |
|  | | |  | | control | | | CD | | | UC | | |  | | |
| 2007 Henckaerts | | | GG | | 263 | | | 797 | | | 234 | | |  | | |
|  | | | GA | | 14 | | | 30 | | | 12 | | |  | | |
|  | | | AA | | 0 | | | 2 | | | 0 | | |  | | |
| 2004 Guo | | | GG | | 110 | | | 15 | | | 47 | | |  | | |
|  | | | GA | | 0 | | | 0 | | | 0 | | |  | | |
|  | | | AA | | 0 | | | 0 | | | 0 | | |  | | |
| 2007 Hong | | | GG | | 177 | | | 176 | | | * | | |  | | |
|  | | | GA | | 11 | | | 6 | | | * | | |  | | |
|  | | | AA | | 0 | | | 0 | | | * | | |  | | |
| 2006 Pierik | | | GG | | 182 | | | 171 | | | 100 | | |  | | |
|  | | | GA | | 9 | | | 8 | | | 6 | | |  | | |
|  | | | AA | | 0 | | | 0 | | | 0 | | |  | | |
| 2010 Shen | | | GG | | 120 | | | 30 | | | 83 | | |  | | |
|  | | | GA | | 0 | | | 0 | | | 0 | | |  | | |
|  | | | AA | | 0 | | | 0 | | | 0 | | |  | | |
| 2009 Lee | | | GG | | 178 | | | 45 | | | 99 | | |  | | |
|  | | | GA | | 0 | | | 0 | | | 0 | | |  | | |
|  | | | AA | | 0 | | | 0 | | | 0 | | |  | | |
| 2011 Chen（1） | | | GG | | 60 | | | 30 | | | 40 | | |  | | |
|  | | | GA | | 0 | | | 0 | | | 0 | | |  | | |
|  | | | AA | | 0 | | | 0 | | | 0 | | |  | | |
| 2011 Chen（2） | | | GG | | 84 | | | 30 | | | 46 | | |  | | |
|  | | | GA | | 0 | | | 0 | | | 0 | | |  | | |
|  | | | AA | | 0 | | | 0 | | | 0 | | |  | | |
| 2007 Xue | | | GG | | 135 | | | 41 | | | 43 | | |  | | |
|  | | | GA | | 0 | | | 0 | | | 0 | | |  | | |
|  | | | AA | | 0 | | | 0 | | | 0 | | |  | | |
|  | | |  | |  | | |  | | |  | | |  | | |
| TLR4 Asp299Gly | | | |  | |  | | |  | | |  | | |  |  |
|  | | | |  | | control | | | CD | | | UC | | |  |  |
| 2007 Henckaerts | | | | AA | | 264 | | | 717 | | | 209 | | |  |  |
|  | | | | AG | | 27 | | | 128 | | | 38 | | |  |  |
|  | | | | GG | | 2 | | | 11 | | | 3 | | |  |  |
| 2004 Guo | | | | AA | | 110 | | | 15 | | | 47 | | |  |  |
|  | | | | AG | | 0 | | | 0 | | | 0 | | |  |  |
|  | | | | GG | | 0 | | | 0 | | | 0 | | |  |  |
| 2005 Braat | | | | AA | | 124 | | | 349 | | | 201 | | |  |  |
|  | | | | AG | | 13 | | | 56 | | | 24 | | |  |  |
|  | | | | GG | | 0 | | | 6 | | | 1 | | |  |  |
| 2005 Oostenbrug | | | | AA | | 269 | | | 343 | | | 159 | | |  |  |
|  | | | | AG | | 27 | | | 47 | | | 19 | | |  |  |
|  | | | | GG | | 0 | | | 3 | | | 1 | | |  |  |
| 2005 Lakatos | | | | AA | | 176 | | | 475 | | | * | | |  |  |
|  | | | | AG | | 23 | | | 50 | | | * | | |  |  |
|  | | | | GG | | 1 | | | 2 | | | * | | |  |  |
| 2005 Gazouli | | | | AA | | 95 | | | 103 | | | 79 | | |  |  |
|  | | | | AG | | 4 | | | 15 | | | 6 | | |  |  |
|  | | | | GG | | 1 | | | 2 | | | 0 | | |  |  |
| 2002 Okayama | | | | AA | | 107 | | | * | | | 86 | | |  |  |
|  | | | | AG | | 0 | | | * | | | 0 | | |  |  |
|  | | | | GG | | 0 | | | * | | | 0 | | |  |  |
| 2004 Arnott | | | | AA | | 157 | | | 186 | | | 212 | | |  |  |
|  | | | | AG | | 31 | | | 46 | | | 32 | | |  |  |
|  | | | | GG | | 1 | | | 2 | | | 2 | | |  |  |
| 2004 Franchimont（1） | | | | AA | | 126 | | | 265 | | | 133 | | |  |  |
|  | | | | AG | | 12 | | | 65 | | | 28 | | |  |  |
|  | | | | GG | | 1 | | | 4 | | | 2 | | |  |  |
| 2004 Franchimont（2） | | | | AA | | 126 | | | 88 | | | * | | |  |  |
|  | | | | AG | | 12 | | | 24 | | | * | | |  |  |
|  | | | | GG | | 1 | | | 1 | | | * | | |  |  |
| 2005 Ouburg | | | | AA | | 153 | | | 364 | | | * | | |  |  |
|  | | | | AG | | 16 | | | 76 | | | * | | |  |  |
|  | | | | GG | | 1 | | | 8 | | | * | | |  |  |
| 2007 Hong | | | | AA | | 158 | | | 156 | | | * | | |  |  |
|  | | | | AG | | 28 | | | 26 | | | * | | |  |  |
|  | | | | GG | | 2 | | | 0 | | | * | | |  |  |
| 2007 Baumgart(1) | | | | AA | | 356 | | | 215 | | | 122 | | |  |  |
|  | | | | AG | | 45 | | | 24 | | | 22 | | |  |  |
|  | | | | GG | | 2 | | | 2 | | | 1 | | |  |  |
| 2007 Baumgart(2) | | | | AA | | 187 | | | 138 | | | 110 | | |  |  |
|  | | | | AG | | 14 | | | 6 | | | 8 | | |  |  |
|  | | | | GG | | 1 | | | 0 | | | 0 | | |  |  |
| 2007 Browning | | | | AA | | 359 | | | 337 | | | 356 | | |  |  |
|  | | | | AG | | 43 | | | 48 | | | 47 | | |  |  |
|  | | | | GG | | 0 | | | 1 | | | 2 | | |  |  |
| 2006 Pierik | | | | AA | | 174 | | | 142 | | | 89 | | |  |  |
|  | | | | AG | | 16 | | | 35 | | | 13 | | |  |  |
|  | | | | GG | | 1 | | | 2 | | | 2 | | |  |  |
| 2006 Figueroa | | | | AA | | 20 | | | 21 | | | 21 | | |  |  |
|  | | | | AG | | 0 | | | 1 | | | 1 | | |  |  |
|  | | | | GG | | 0 | | | 0 | | | 0 | | |  |  |
| 2013 Manolakis | | | | AA | | 242 | | | 166 | | | 127 | | |  |  |
|  | | | | AG | | 31 | | | 21 | | | 36 | | |  |  |
|  | | | | GG | | 1 | | | 0 | | | 0 | | |  |  |
| 2012 Sivaram | | | | AA | | 153 | | | * | | | 107 | | |  |  |
|  | | | | AG | | 23 | | | * | | | 30 | | |  |  |
|  | | | | GG | | 0 | | | * | | | 2 | | |  |  |
| 2010 Shen | | | | AA | | 120 | | | 30 | | | 83 | | |  |  |
|  | | | | AG | | 0 | | | 0 | | | 0 | | |  |  |
|  | | | | GG | | 0 | | | 0 | | | 0 | | |  |  |
| 2010 Wagner | | | | AA | | 84 | | | 59 | | | * | | |  |  |
|  | | | | AG | | 12 | | | 11 | | | * | | |  |  |
|  | | | | GG | | 2 | | | 2 | | | * | | |  |  |
| 2009 Ye | | | | AA | | 336 | | | 360 | | | * | | |  |  |
|  | | | | AG | | 0 | | | 0 | | | * | | |  |  |
|  | | | | GG | | 1 | | | 0 | | | * | | |  |  |
| 2009 Zouiten-Mekki | | | | AA | | 71 | | | 78 | | | * | | |  |  |
|  | | | | AG | | 9 | | | 12 | | | * | | |  |  |
|  | | | | GG | | 0 | | | 0 | | | * | | |  |  |
| 2009 Queiroz | | | | AA | | 489 | | | 41 | | | 38 | | |  |  |
|  | | | | AG | | 50 | | | 2 | | | 3 | | |  |  |
|  | | | | GG | | 0 | | | 0 | | | 1 | | |  |  |
| 2009 Bueno | | | | AA | | 71 | | | 66 | | | 15 | | |  |  |
|  | | | | AG | | 8 | | | 11 | | | 0 | | |  |  |
|  | | | | GG | | 0 | | | 3 | | | 0 | | |  |  |
| 2008 Rigoli | | | | AA | | 95 | | | 123 | | | 45 | | |  |  |
|  | | | | AG | | 8 | | | 10 | | | 42 | | |  |  |
|  | | | | GG | | 0 | | | 0 | | | 3 | | |  |  |
| 2008 Hume | | | | AA | | 327 | | | 533 | | | * | | |  |  |
|  | | | | AG+GG | | 33 | | | 86 | | | * | | |  |  |
| 2009 Lee | | | | GG | | 178 | | | 45 | | | 99 | | |  |  |
|  | | | | GA | | 0 | | | 0 | | | 0 | | |  |  |
|  | | | | AA | | 0 | | | 0 | | | 0 | | |  |  |
| 2011 Chen（1） | | | | GG | | 60 | | | 30 | | | 40 | | |  |  |
|  | | | | GA | | 0 | | | 0 | | | 0 | | |  |  |
|  | | | | AA | | 0 | | | 0 | | | 0 | | |  |  |
| 2011 Chen（2） | | | | GG | | 84 | | | 30 | | | 46 | | |  |  |
|  | | | | GA | | 0 | | | 0 | | | 0 | | |  |  |
|  | | | | AA | | 0 | | | 0 | | | 0 | | |  |  |
| 2007 Jiang | | | | GG | | 150 | | | * | | | 68 | | |  |  |
|  | | | | GA | | 0 | | | * | | | 0 | | |  |  |
|  | | | | AA | | 0 | | | * | | | 0 | | |  |  |
| 2007 Xue | | | | GG | | 135 | | | 41 | | | 43 | | |  |  |
|  | | | | GA | | 0 | | | 0 | | | 0 | | |  |  |
|  | | | | AA | | 0 | | | 0 | | | 0 | | |  |  |
|  | | | |  | |  | | |  | | |  | | |  |  |
| TLR4 Thr399Ile |  | | | | |  | | |  | | |  | | |  | |
|  |  | | | | | control | | | CD | | | UC | | |  | |
| 2004 Guo | CC | | | | | 110 | | | 15 | | | 47 | | |  | |
|  | CT | | | | | 0 | | | 0 | | | 0 | | |  | |
|  | TT | | | | | 0 | | | 0 | | | 0 | | |  | |
| 2012 Azzam | CC | | | | | 32 | | | 24 | | | * | | |  | |
|  | CT | | | | | 14 | | | 18 | | | * | | |  | |
|  | TT | | | | | 4 | | | 4 | | | * | | |  | |
| 2005 Oostenbrug | CC | | | | | 270 | | | 440 | | | 197 | | |  | |
|  | CT | | | | | 29 | | | 59 | | | 22 | | |  | |
|  | TT | | | | | 0 | | | 5 | | | 1 | | |  | |
| 2005 Gazouli | CC | | | | | 2 | | | 119 | | | 82 | | |  | |
|  | CT | | | | | 0 | | | 1 | | | 3 | | |  | |
|  | TT | | | | | 1 | | | 0 | | | 0 | | |  | |
| 2007 Hong | CC | | | | | 158 | | | 152 | | | * | | |  | |
|  | CT | | | | | 28 | | | 30 | | | * | | |  | |
|  | TT | | | | | 2 | | | 0 | | | * | | |  | |
| 2007 Browning | CC | | | | | 347 | | | 339 | | | 348 | | |  | |
|  | CT | | | | | 46 | | | 45 | | | 45 | | |  | |
|  | TT | | | | | 0 | | | 1 | | | 1 | | |  | |
| 2013 Manolakis | CC | | | | | 242 | | | 166 | | | 127 | | |  | |
|  | CT | | | | | 31 | | | 21 | | | 36 | | |  | |
|  | TT | | | | | 1 | | | 0 | | | 0 | | |  | |
| 2010 Shen | CC | | | | | 120 | | | 30 | | | 83 | | |  | |
|  | CT | | | | | 0 | | | 0 | | | 0 | | |  | |
|  | TT | | | | | 0 | | | 0 | | | 0 | | |  | |
| 2009Zouiten-Mekki | CC | | | | | 72 | | | 77 | | | 28 | | |  | |
|  | CT | | | | | 8 | | | 13 | | | 2 | | |  | |
|  | TT | | | | | 0 | | | 0 | | | 0 | | |  | |
| 2009 Lee | CC | | | | | 178 | | | 45 | | | 99 | | |  | |
|  | CT | | | | | 0 | | | 0 | | | 0 | | |  | |
|  | TT | | | | | 0 | | | 0 | | | 0 | | |  | |
| 2011 Chen（1） | CC | | | | | 60 | | | 30 | | | 40 | | |  | |
|  | CT | | | | | 0 | | | 0 | | | 0 | | |  | |
|  | TT | | | | | 0 | | | 0 | | | 0 | | |  | |
| 2011 Chen（2） | CC | | | | | 84 | | | 30 | | | 46 | | |  | |
|  | CT | | | | | 0 | | | 0 | | | 0 | | |  | |
|  | TT | | | | | 0 | | | 0 | | | 0 | | |  | |
| 2007 Xue | GG | | | | | 135 | | | 41 | | | 43 | | |  | |
|  | GA | | | | | 0 | | | 0 | | | 0 | | |  | |
|  | AA | | | | | 0 | | | 0 | | | 0 | | |  | |
|  |  | | | | |  | | |  | | |  | | |  | |
