# Supplementary material for: Association between TLR2 and TLR4 Gene Polymorphisms and the Susceptibility to Inflammatory Bowel Disease: A Meta-Analysis
Source: PLoS One. 2015 May 29;10(5):e0126803. doi: 10.1371/journal.pone.0126803 (PMC4449210; doi:10.1371/journal.pone.0126803)
Supplement: S2 Table — (DOC) [file pone.0126803.s003.doc]

**Table S2. Studies referring TLR2/TLR4 polymorphism and IBD susceptibility, but not meet the inclusion criteria for the meta-analysis**

|  |  |  |
| --- | --- | --- |
| **Author, year of Publication** | **Title of the excluded studies** | **Reason for exclusion** |
| Braat, H., 2003 | A FUNCTIONAL SINGLE NUCLEOTIDE POLYMORPHISM OF THE TLR4 GENE IS CORRELATED | Overlap with Braat 2005[31] |
|  | WITH CROHN'S DISEASE BUT NOT WITH ULCERATIVE COLITIS. |  |
| Oostenbrug, L.E.,2003 | LACK OF ASSOCIATION BETWEEN TOLL-LIKE RECEPTOR 4 HAPLOTYPE AND ASP299GLY | Overlap with Oostenbrug 2005[32] |
|  | POLYMORPHISM AND SUSCEPTIBILITY FOR INFLAMMATORY BOWEL DISEASE. |  |
| McGovern, D.,2003 | INNATE IMMUNOGENETICS AND INFLAMMATORY BOWEL DISEASE (IBD). | Not sufficient data for genetype |
|  |  |  |
| Guo, 2004 | Association between Toll-like Receptor-4 Gene Polymorphism and Inflammatory Bowel | Overlap with Xiong 2007[36] |
|  | Disease and Colorectal Cancer |  |
| Mallant-Hent, R., 2005 | The Toll-like receptor 4 (tlr4) Asp299gly polymorphism is associated with colonic | Overlap with Ouburg 2005[30] |
|  | localization of Crohn's disease, without a major role for the Saccharomyces |  |
|  | cerevisiae Mannan-Lbp-Cdl4-Tlr4 pathway. |  |
| Wells GA, S.B.O.C., 2005 | Toll-like receptor 4 and NOD2/CARD15 mutations in Hungarian patients with Crohn disease: | Overlap with Lakatos 2005[33] |
|  | Phenotype-genotype correlations. |  |
| Guo, Q.S.,2005 | Polymorphisms of CD14 gene and TLR4 gene are not associated with ulcerative colitis in | Overlap with Xiong 2007[36] |
|  | Chinese patients. |  |
| Hume, G.E.,2005 | The role of TLR4 Asp299Gly and TLR5 Arg392Stop in an Australian inflammatory bowel | Overlap with Hume 2008[44] |
|  | disease cohort. |  |
| Bueno De Mesquita, M.,2005 | Study on CARD15 and TLR4 polymorphisms in children with early diagnosed IBD and their | Overlap with Bueno 2009[50] |
|  | family members. |  |
| Henckaerts, L., 2006 | Mutations in innate immune receptors modulate the serologic response to microbial | Overlap with Henckaerts 2007[29] |
|  | antigens in patients with inflammatory bowel disease. |  |
| Li-Fen Xiong,2006 | No association of TLR4 gene Asp299Gly,TLR2gene Arg753Glu and Arg677Trp Polymorphisms | Overlap with Xiong 2007[36] |
|  | bowel disease in Chinese Han Population of Hubei Province |  |
| Jiangyi,2007 | The association between Toll- like receptor- 4 gene Asp299Gly polymorphism and ulcerative | Overlap with Jiang 2006[37] |
|  | colitis and colorectal adenocarcinoma. |  |
| Lappalainen, M.,2008 | Association of IL23R, TNFRSF1A, and HLA-DRB1*0103 allele variants with inflammatory | Not sufficient data for genetype |
|  | bowel disease phenotypes in the Finnish population. |  |
| Mendoza, J.L.,2008 | Innate immune defects in IRGM1, TLR2, and NOD2 contribute to Crohn's disease risk and | Not sufficient data for genetype |
|  | severity in Ashkenazi Jews. |  |
| Zouiten-Mekki L.,2009 | Tolllike receptor 4 (TLR4) polymorphisms in Tunisian patients with Crohn's disease: | Overlap with Zouiten-Mekki 2009[48] |
|  | Genotype-phenotype correlation |  |
| Magalhäes Queiroz D.M.,2009 | Immune response and gene polymorphism profiles in Crohn's disease and ulcerative colitis | Overlap with Queiroz 2009[49] |
|  |  |  |
| Lee, K, 2009 | A Polymorphism of CD14 Is Associated with Inflammatory Bowel Disease in Korean Population | Overlap with Kim 2012[56] |
| Shen X.-Y, 2010 | Toll- like receptor gene polymorphisms and susceptibility to inflammatory bowel disease in | Overlap with Shen 2010[52] |
|  | Chinese Han and Caucasian populations |  |
| Shen, X, 2010 | The Toll-like receptor 4 D299G and T399I polymorphisms are associated with Crohn's disease | Overlap with Shen 2010[52] |
|  | and ulcerative colitis: a meta-analysis. |  |
| Chen L., 2012 | Analysis of TLR4 and TLR2 polymorphisms in inflammatory bowel disease in a Guangxi Zhuang population. | Overlap with Chen 2011[53] |
|  |  |  |
| Sivaram, G.,2012 | Macrophage migration inhibitory factor, Toll-like receptor 4, and CD14 polymorphisms with | Overlap with Sivaram 2012[54] |
|  | altered expression levels in patients with ulcerative colitis. |  |
| Manolakis, A.C.A.Y.,2013 | Readressing the Role of Toll-Like Receptor-4 Alleles in Inflammatory Bowel Disease: | Overlap with Manolakis[58] |
|  | Colitis, Smoking, and Seroreactivity. |  |
|  |  |  |
|  |  |  |
